# Treating suicidal ideation in the context of depression

**DOI:** 10.1186/s12888-020-02894-5

**Published:** 2020-10-08

**Authors:** Renee A. Schneider, Shih Yin Chen, Anita Lungu, Joseph R. Grasso

**Affiliations:** Lyra Health, Burlingame, USA

**Keywords:** Empirically-supported treatment, Depression, Suicidal ideation

## Abstract

**Background:**

Treatment recommendations suggest that suicidal ideation will decrease following successful psychotherapy for depression. However, findings from the empirical research are equivocal in this regard. It is possible suicidal ideation does not respond to empirically supported treatment (EST) for depression or that suicidal ideation limits the efficacy of ESTs for depression.

**Methods:**

Data from 793 patients who sought EST for depression was analyzed using *t*-tests and multiple linear regression.

**Results:**

Both patients with (*n* = 233) or without suicidal ideation (*n* = 560) were significantly less depressed following treatment. A significant reduction in suicidal ideation was also observed. At baseline, 233 (29.4%) patients reported suicidal ideation, whereas only 90 (11.3%) patients reported suicidal ideation at follow-up. The relationship between suicidal ideation at baseline and depression scores at follow-up was not significant.

**Conclusions:**

Patients with suicidal ideation who receive short-term EST can experience significant reductions in both depressive symptoms and suicidal ideation. Findings suggest that suicidal ideation at baseline does not impact treatment efficacy, but additional research that directly tests moderation is needed.

## Background

Suicidal ideation is common among people with mental health problems, particularly depression [1, 2]. According to the Substance Abuse and Mental Health Services Administration, nearly 30% of people experiencing depression also have thoughts of suicide [3]. Despite the overlap between depression and suicidal ideation, there are no recognized short-term empirically supported treatments (ESTs) for managing suicidal ideation in the context of depression. ESTs are defined as behavioral health interventions that have been rigorously tested in randomized controlled trials or a series of well-designed single subject experiments and have demonstrated efficacy when compared to a control or active treatment condition [4, 5]. ESTs are generally short-term, manualized interventions for specific disorders [6]. Although treatment guidelines for patients with depression and suicidal ideation suggest that ESTs aimed at reducing depressive symptomatology will also reduce suicidal thoughts and behaviors [7], there is little empirical research supporting this assumption [8].

In their review of the research, Cuijpers and colleagues [8] were able to identify only three randomized controlled trials that tested the efficacy of short-term psychotherapy for depression on suicidal ideation. Overall, results demonstrated that short-term psychotherapy for depression had little to no appreciable effect on suicidal ideation. However, limited statistical power and methodological limitations led Cuijpers et al. [8] to conclude that more research is needed to determine if psychotherapy aimed at depression is also effective in reducing suicidal thoughts. A later study by Weitz, Hollon, Kerkhof, and Cuijpers [9] utilized data from the Treatment Depression Research Collaborative to test whether short-term ESTs for depression were effective in treating suicidal ideation. Results revealed that interpersonal psychotherapy (IPT [10];), but not cognitive behavioral therapy (CBT [11];), reduced rates of suicidal ideation among patients with depression. However, in a more recent study of veterans receiving CBT, rates of suicidality decreased significantly following treatment [12].

Given limited research and conflicting findings in this area, we do not know whether ESTs are effective in reducing suicidal ideation. In addition, there is a paucity of research examining the impact of suicidal ideation on treatment efficacy for depression. It is possible that persistent thoughts of wanting to end one’s life or the chronic hopelessness or despair that often accompany these thoughts may diminish the effectiveness of ESTs. The present study, therefore, was designed to further our understanding of the impact of short-term ESTs on suicidal ideation in the context of depression. We were interested in learning whether short-term ESTs are effective in treating both depression and suicidal ideation, among patients with these commonly comorbid conditions. We were also interested in whether suicidal ideation at baseline is associated with poorer treatment outcomes.

## Methods

### Participants

Adult patients, age 18 or older, who started individual therapy between July 1, 2018 and May 31, 2019 were included in the present study. All patients in the study were referred to a community therapist by an employee assistance program (EAP) that partners with therapists who utilize ESTs. Patients were employees or dependents of employers who contract with the EAP. Patients were included in the study if they scored in the clinical range on a measure of depression and completed a baseline assessment within 2 weeks of their first appointment. Patients were sent electronically secure assessment questionnaires every 4 weeks following their first appointment. Baseline assessments were compared to the most recent assessment to estimate the impact of treatment. This study was deemed exempt from human patients review by the Western Institutional Review Board (WIRB). Informed consent was not collected, as this study involved a retrospective review of clinical records.

### Therapists

All therapists in the present study were in community private or group practices and had agreed to join the network of an EAP that specializes in referring clients to providers who practice ESTs. Prior to joining the EAP network, each provider was asked to complete a written application, noting which psychotherapy treatments they utilized. This along with their professional online presence (e.g., private practice website) was reviewed to determine whether the provider likely utilized ESTs. Those providers who passed this initial step were invited to participate in a phone-based, clinical interview designed to test their knowledge of ESTs and ability to apply these therapies in clinical practice. Sample components of the clinical interview include asking prospective therapists about their theoretical orientation, the therapies and interventions they use, how they measure treatment progress, the average length of treatment, and how they adapt treatment plans based on a patient’s response to treatment. Only those therapists who passed the rigorous clinical vetting interview were invited to join the network. Historically, 5% of providers have passed the review and vetting interview. The types of therapies providers accepted into the network endorsed include Cognitive-Behavioral Therapy [11], Dialectical Behavior Therapy [13], Emotion-Focused Therapy [14], and Interpersonal Psychotherapy [10].

### Measures

Patients were included in the study dataset if they scored in the clinical range on a measure of depression and completed a baseline assessment within 2 weeks of their first appointment. Patients were sent electronically secure assessment questionnaires every 4 weeks after their first appointment with a provider. Baseline assessments were compared to the most recent assessment to estimate the impact of treatment.

The PHQ-9 was used to assess depression symptoms [15]. The PHQ-9 includes 9 items rated on a 4-point scale, with scores ranging from 0 to a maximum of 27. For inclusion in the study, a clinical cut-off of 10 was used for the PHQ-9, as research suggests that patients who score at or above 10 are very likely to meet criteria for major depression [15]. Item 9 of the PHQ-9 was used to assess suicidal ideation (i.e., thoughts of hurting or killing oneself). Scores on this item ranged from 0 to 3, with 3 indicating more frequent thoughts of suicide. In analyses, to separate the effects of depression from suicidal ideation, we used only the first 8 items of the PHQ-9 (i.e., the PHQ-8) as a measure of depression severity, consistent with the methodology used in previous research [9].

### Analyses

We used paired *t*-tests to examine changes in depression and suicidal ideation from baseline to follow-up. Cohen’s *d* is reported as a measure of effect size. We also report rates of clinically significant improvement, calculated as a decrease of at least 5 points on the PHQ-8 [16], for patients with or without suicidal ideation at baseline to determine whether short-term ESTs are effective in reducing depressive symptoms, even among patients with suicidal ideation. Multiple linear regression was used to test whether suicidal ideation at baseline was associated with higher levels of depression at follow-up, after controlling for depression severity at baseline.

## Results

Of the 793 patients included in analyses, 60.7% (*n* = 481) identified as female, 39.3% (*n* = 312) identified as male. The mean age of patients was 32.8 years (*SD =* 9.0). The 793 patients saw 452 therapists. On average, each therapist saw 1.8 (*SD =* 1.2) patients. Approximately 43.6% (*n* = 197) of therapists had a doctoral degree; 56.4% (*n* = 255) of therapists had a master’s degree (e.g., LMFT, LCSW, LPCC) (Table 1).

**Table 1:** Participant and provider characteristics

	Full sample	PHQ suicide > 0
**Clients**		
N	793	233
Gender		
Female (%)	60.7	55.8
Age		
Mean (SD)	32.8 (9.0)	31.2 (8.0)
**Therapists**		
N	452	179
Therapist Credential		
Doctoral-level (%)	43.6	44.7
Years of Experience		
< 5 years (%)	25.2	24.6
5–10 years (%)	35.2	43.0
10–15 years (%)	20.4	16.8
15–20 years (%)	10.0	10.1
> 20 years (%)	9.3	5.6

Therapists delivered ESTs, as per their normal practice, and patients could receive up to a pre-specified number of session visits per calendar year, depending on the benefit design offered by the employer, up to 25 sessions or 6 months of treatment. The average number of sessions delivered across the course of treatment was 8.7 (*SD* = 5.7). The average number of weeks clients spent in treatment was 12.0 (*SD* = 8.8). Sessions were typically 45–50 min in length.

Of the 793 patients who scored in the clinical range on the PHQ-9 at baseline, 560 (70.6%) reported no suicidal ideation (item 9 = 0); 184 (23.2%) had ideation several days in the past week (item 9 = 1); 33 (4.2%) indicated they had ideation more than half the days in the past week (item 9 = 2), and 16 (2.0%) indicated they had ideation nearly every day in the past week (item 9 = 3) (Table 2). No statistically significant differences in baseline suicidal ideation status were found by gender (χ^2^ (3) = 4.3, *p* = 0.2). The suicide item was weakly correlated (*r* (791) = .32, *p* < .001) with the depression score (PHQ-8).

**Table 2:** PHQ suicidal ideation item frequencies at the baseline and final assessment (*n* = 793)

Thoughts that you would be better off dead or of hurting yourself in some way	Baseline (%)	Follow-up (%)
0. Not at all	560 (70.6)	703 (88.7)
1. Several days	184 (23.2)	76 (9.6)
2. More than half the days	33 (4.2)	8 (1.0)
3. Nearly every day	16 (2.0)	6 (0.8)

*PHQ* = Patient Health Questionnaire

For patients who started with no suicidal ideation, the mean PHQ-8 score decreased significantly by an average of 6.0 points (*SD* = 4.4, *t*(559) = 31.3, *p* < .001), from 13.4 (*SD* = 3.1) at baseline to 7.4 (*SD* = 4.0) at follow-up. Results of paired *t*-test indicated that for patients with any suicidal ideation at baseline (i.e., a score > 0 on item 9 of the PHQ-9), the mean depression score on the PHQ-8 decreased significantly, from 15.3 (*SD* = 4.1) at baseline to 7.9 (*SD* = 5.1) at follow-up, with patients improving an average of 7.4 points (*SD* = 5.5, *t*(232) = 20.7, *p* < .001). Cohen’s *d* suggested large effect sizes for both groups with and without suicidal ideation, *d* = 1.59 and *d* = 1.69, respectively. (Table 3). Clinically significant change (5-point or greater change) in PHQ-8 was observed in 63.4% of patients with no suicidal ideation at baseline and 71.2% of patients with suicidal ideation at baseline.

**Table 3:** Baseline and follow-up means, standard deviations, and effect sizes for PHQ-8 and suicide item (SI) by group

Measure	N	Baseline M (SD)	Follow-up M (SD)	Paired differences M (SD)	t-test	Cohen’s d
**PHQ-8**						
SI > 0 at baseline	233	15.3 (4.1)	7.9 (5.1)	7.4 (5.5)	*t*(232) = 20.7, *p* < .001	1.59
SI = 0 at baseline	560	13.4 (3.1)	7.4 (4.0)	6.0 (4.4)	*t*(559) = 31.3, *p* < .001	1.69
**Suicide item**						
SI > 0 at baseline	233	1.3 (0.6)	0.3 (0.6)	0.9 (0.7)	*t*(232) = 21.3, *p* < .001	1.54

Of the 233 patients who had any suicidal ideation at baseline, the mean suicide score was 1.3 (*SD* = 0.6) at baseline and 0.3 (SD = 0.6) at follow-up. Results of paired *t*-test revealed that the suicide score decreased significantly between baseline and follow-up, with an average improvement of 0.9 points (*SD* = 0.7, *t*(232) = 21.3, *p* < .001) (Table 3). Cohen’s *d* suggested a large treatment effect size on suicide score (*d* = 1.5). Of the 233 patients with suicidal ideation at baseline, 73.0% reported no suicidal ideation at follow-up; of the 560 patients with no suicidal ideation at baseline, 4.8% of them reported suicidal ideation at follow-up.

Results from regression analysis, controlling for age, gender, and session count, indicated that patients with higher PHQ-8 scores at baseline had higher PHQ-8 scores at follow-up (*β* = 0.33, *SE* = 0.04, *t* = 7.62, *p* < .001). However, suicidal ideation at baseline did not predict PHQ-8 score at follow-up (*β* = − 0.08, *SE* = 0.34, *t* = − 0.23, *p* = 0.82), after controlling for confounding variables.

## Discussion

Overall, findings suggest that short-term ESTs can be effective in treating suicidal ideation in the context of depression, as suicidal ideation decreased significantly following treatment with an EST. In addition, both patients with or without suicidal ideation demonstrated clinically significant changes in depression severity, suggesting that suicidal ideation is not necessarily an impediment to treatment efficacy. However, all of the patients in this study were outpatients and most had only infrequent suicidal ideation. In fact, it may be that depression severity was only weakly correlated with suicidal ideation because there was little variability in reports of suicidal ideation. For this reason, the finding that suicidal ideation does not interfere with treatment for depression, should be interpreted cautiously, as more frequent or severe suicidal ideation could interfere with treatment. Overall, however, our findings support treatment recommendations that direct patients with suicidal ideation and depression to short-term ESTs for depression [7].

Many clinicians express a hesitancy or unwillingness to treat patients with suicidal ideation [17]. Patients with suicidal thoughts are often excluded from randomized controlled trials [18], and therapists in the community may turn away patients who indicate they are having thoughts of hurting or killing themselves. Therapists may be reluctant to treat patients with suicidal ideation because of the inherent risk in doing this work. Certainly, no clinician in academia or the community wants to manage the aftermath of a patient suicide. However, therapists may also feel ill-equipped to treat patients with suicidal ideation [19], particularly given that no short-term ESTs for addressing suicidal ideation currently exist. Findings presented here provide a counterpoint to these concerns, as the evidence suggests that ESTs for depression can effectively reduce suicidal ideation.

A number of limitations to this study should be noted. First, we cannot determine from this study which ESTs are most effective, as patient records did not indicate which specific ESTs were used. Previous research supports the use of IPT and some research supports the use of CBT, but additional research is needed to confirm these findings and to determine whether other ESTs, are also effective in reducing suicidal ideation in the context of depression. Second, our measure of suicidality was narrow and only allowed us to look at suicidal ideation; we did not have data on which patients had a previous or recent suicide attempt, the age of onset of depression, or the number of previous episodes. Future studies are needed to determine whether ESTs are effective in reducing not only thoughts of suicide but suicide attempts, as well. Additional research should also focus on whether ESTs are less potent for patients with early-onset or recurrent depression, relative to those with a single episode. Because patients in this study were relatively young, it is also unclear whether findings will generalize to older patients or those with more chronic depression.

This study is also limited because we did not have data on patients’ formal diagnoses. It is possible that ESTs are effective in ameliorating suicidal ideation among patients with some depressive disorders, but not others. Although assessments were administered at regular intervals (once every month), patients completed follow-up assessments irregularly, and it is possible that some patients completed the last assessment prior to the end of therapy. Had all patients completed the last assessment at the end of treatment, rates of depression and suicidality may have been even lower.

It would be helpful to know whether short-term ESTs exert a direct effect on suicidal ideation or influence it indirectly by lowering the severity of overall depression. Research on how ESTs affect other variables that may moderate (e.g., chronic health problems, relationship problems) or mediate (e.g., emotion regulation strategies, hopelessness) relations between depression and suicidality is also needed. Finally, because everyone in the current study received short-term outpatient treatment, it was impossible to directly test whether suicidal ideation moderates the efficacy of treatment for depression. Ideally, research would involve randomizing patients to different treatment conditions to see if suicidal ideation is associated with greater levels of depression post-treatment.

## Conclusion

Despite these limitations, the present study provides much needed data regarding the outcomes of patients with suicidal ideation who receive ESTs for depression in the community. With millions of people experiencing suicidal ideation, it is critical that further studies on psychotherapy outcomes are conducted to inform clinical practice guidelines and ultimately, ease the intense suffering of patients with suicidal ideation. In addition, it is vital that we begin to address the question of whether short-term ESTs for depression are effective in reducing rates of attempted and completed suicides.

## Data Availability

The data that used was provided by a third party, Lyra Clinical Associates P.C. While having the rights to share the data for research, Lyra Clinical Associates P.C., treats the data as highly confidential and does not make such data available to the public. The data would be available from the authors upon a reasonable request, and with permission of Lyra Clinical Associates provided that any requestor agrees to keep the data restricted.

## Abbreviations

CBT: Cognitive behavioral therapy
EST: Empirically supported treatment
EAP: Employee assistance program
IPT: Interpersonal psychotherapy
DBT: Dialectical behavior therapy
EFT: Emotion-focused therapy
WIRB: Western Institutional Review Board
